# Excess pneumonia and influenza hospitalizations associated with influenza epidemics in Portugal from season 1998/1999 to 2014/2015

**DOI:** 10.1111/irv.12501

**Published:** 2018-02-19

**Authors:** Emanuel Rodrigues, Ausenda Machado, Susana Silva, Baltazar Nunes

**Affiliations:** ^1^ Departamento de Epidemiologia Instituto Nacional de Saúde Dr. Ricardo Jorge Lisboa Portugal; ^2^ Escola Nacional de Saúde Pública Universidade NOVA de Lisboa Lisboa Portugal

**Keywords:** autoregressive integrated moving average models, excess hospitalizations, influenza epidemics, vaccine coverage

## Abstract

**Background:**

The aim of this study was to estimate excess pneumonia and influenza (P&I) hospitalizations during influenza epidemics and measure their correlation with influenza vaccine coverage in the 65 and more years old, according to the type/subtype of influenza virus.

**Methods:**

The study period comprised week 40/1998‐40/2015. Age‐specific weekly P&I hospitalizations (ICD‐9: 480‐487) as main diagnosis were extracted from the National Hospital Discharge database. Age‐specific baseline hospitalization rates were estimated by autoregressive integrated moving average (ARIMA) model without time periods with excess hospitalizations. Excess hospitalizations were calculated by subtracting expected hospitalization rates from the observed during influenza epidemic periods. Correlation between excess P&I hospitalizations and influenza vaccine coverage in the elderly was measured with Pearson correlation coefficient.

**Results:**

The average excess P&I hospitalizations/season was 19.4/10^5^ (range 0‐46.1/10^5^), and higher excess was observed in young children with <2 years (79.8/10^5^) and ≥65 years (68.3/10^5^). In epidemics with A(H3) dominant, the highest excess hospitalizations were observed among 65 and over. Seasons which influenza B or A(H1)pdm09 dominance the highest excess was observed in children with <2 years. High negative correlation was estimated between excess hospitalizations associated with A(H3) circulation and vaccine coverage in the elderly (*r* = −.653; 95% CI: −0.950 to −0.137).

**Conclusion:**

Over 80% of the influenza epidemics were associated with excess hospitalizations. However, excess P&I hospitalizations pattern differed from age group and circulating virus. This ecologic approach also identified a reduction in excess P&I associated with A(H3) circulation with increasing vaccine coverage in the elderly.

## 1 INTRODUCTION

Influenza viruses circulate every year, causing epidemics that are usually benign and mild for the human population but that can complicate into other diseases, like pneumonia. According to Wuerth et al (2016),^1^ in the 2002‐2011 period, influenza was the fourth causative agent for pneumonia and approximately 9/100 000 of pneumonia hospitalizations had influenza as the etiological respiratory agent. However, as influenza laboratory diagnosis is not usually performed in all suspected cases, using these data underestimate the influenza impact.^2^, ^3^ Taking this into consideration, the overall effect of influenza epidemics has been measured through indirect ecologic methods using the Serfling approach^4^ and Poisson, negative binomial regression and autoregressive integrated moving average (ARIMA) models to estimate influenza‐associated mortality or hospitalizations rates.^5^, ^6^, ^7^, ^8^, ^9^ There are two main approaches in estimating influenza‐associated excesses: one based on statistical models that include influenza activity indicators as explanatory covariates; another does not consider covariates and by excluding from the estimating process all parts of the outcome time series where there is evidence of occurrence of some event that might influence the outcome.^9^ Both approaches have pros and cons. Using models with covariates allows estimating influenza‐associated outcomes by virus type and subtype, but requires robust virological data.^10^ In the alternative, this specific data requirement is not needed and can be used provided that consistent mortality or hospitalization time series are available.^10^ However, there are limitations also in this approach. The lack of virological covariates in the model implies the assumption that all excess winter mortality is associated with influenza circulation which may not be appropriate, leading therefore to a mortality overestimation.

The identification of influenza epidemics requires influenza surveillance data, with information on influenza virus type, and influenza epidemic activity period.^11^, ^12^ Also, and when available, the identification of other events that contribute to mortality or hospitalizations distribution, like secular trend or seasonality, is desirable so to get a better fit of the model to the time series and improve the quality and the validity of the influenza attributable excess estimate.^13^

The influenza impact is particularly evident in specific groups, like the elderly, pregnant and those with chronic disease, with higher risk of complications associated with influenza infection leading to hospitalization or death.^14^ For this high‐risk group of individuals, yearly vaccination in the autumn is recommended in Portugal and in most EU countries, with the intention of reducing risk of complications, severe disease, and death.^15^, ^16^ The influenza vaccine has proven to be moderately effective in reducing medically attended confirmed influenza.^17^ Using an ecologic approach, one would expect a reduction in excess hospitalizations/mortality with increasing vaccine coverage and this was already reported in previous influenza‐related outcomes studies.^18^, ^19^

In Portugal, there are several studies that associate this respiratory infection with excess pneumonia and influenza (P&I) and all‐causes mortality.^8^, ^13^ However, information on influenza impact on morbidity indicators, such as hospital admissions, is scarce and the knowledge of influenza impact and the role of immunization on hospitalizations is essential for a better resource management and for preparing mitigation measures.

Considering the Portuguese mainland context, this study aims to (i) estimate the excess number of P&I hospitalizations during influenza epidemics from seasons 1998‐1999 to 2014‐2015 and (ii) to measure their correlation with influenza vaccine coverage in the elderly (65 and more years old), according to the type/subtype of influenza virus predominant in each season.

## 2 METHODS

A time series ecological study was conducted to estimate the baseline of weekly P&I hospitalizations free of influenza epidemics and estimate excess P&I hospitalizations associated with influenza epidemics between 1998/1999 and 2014/2015 seasons.

### 2.1 Hospital discharge data

Weekly hospitalizations with P&I as principal diagnosis, according to the International Classification of Disease (ICD 9: 480‐487), were extracted from the National Hospital Discharge database (1998‐2015). Weekly time series were disaggregated by age group: <2, 2‐4, 5‐14, 15‐49, 50‐64, and 65 and more years.

### 2.2 Influenza activity

The definition of influenza epidemic periods was based on information from the Portuguese Influenza Surveillance System. The influenza epidemic period, according information from the Portuguese Influenza Surveillance System, was defined as the period with weekly estimates of influenza‐like illness (ILI) incidence rates obtained by the Portuguese General Practitioner (GP) Sentinel Network (Rede Médicos‐Sentinela) above the baseline with non‐sporadic detections of influenza viruses.^20^ Taking this into consideration, an influenza epidemic was identified in all seasons of the study period, exception for the 2005/2006 season. One week was added at the end of the influenza epidemic period to account for eventual delays on the impact.

Predominant virus circulating was provided by Portuguese Laboratory Network for the Diagnosis of Influenza Infection, namely by National Influenza Reference Laboratory, and was defined according to Influenza Reporting Protocol.^21^ The threshold for dominance was set at 60%, and the threshold for codominance is set between 40% and 60%.^21^

### 2.3 Periods potentially associated with excess hospitalizations

These periods include the influenza epidemic (description above on Influenza activity), 2009 pandemic influenza,^22^ and heat wave periods (weeks in which two or more consecutive days had an average maximum daily temperature above 32°C with an extra week to account the known delay of impact.^13^, ^23^

### 2.4 Influenza vaccine coverage rates

The influenza vaccine coverage (IVC) rates, for individuals with 65 and more years, were obtained from ECOS (Em Casa Observamos Saúde—At Home We Watch Health), a panel of approximately 1000 households on which a seasonal survey is carried out by computer‐assisted telephone interview (CATI).^24^ These households were selected randomly from the national telephone directory and recruited considering the representativeness of Portuguese mainland families reachable by telephone.

### 2.5 Methods to estimate the number of excess hospitalization associated with influenza epidemics

Age‐specific baseline hospitalization rates were estimated by ARIMA model,^25^ after extracting from the time series the periods potentially associated with excess hospitalizations (defined above and presented on Table S1), using Flubase R package.^26^ ARIMA model is composed of 3 terms: first, an autoregressive term (AR), in which the time series is regressed on itself at specific lag times; second, a moving average term (MA), in which the time series is regressed on the regression errors at specific lag times; and finally, an integration term that accounts for the non‐stationarity of the time series. If the integrated component is present, the time series is differentiated on itself at specific lag periods; if I = 1, the original time series is transformed in new time series = y(t)−y(t−1).

Using an automatic model identification algorithm included on the package, which recurs to a specific R package named forecast, the final models (Table S2) were selected.^27^

**Figure 1:**
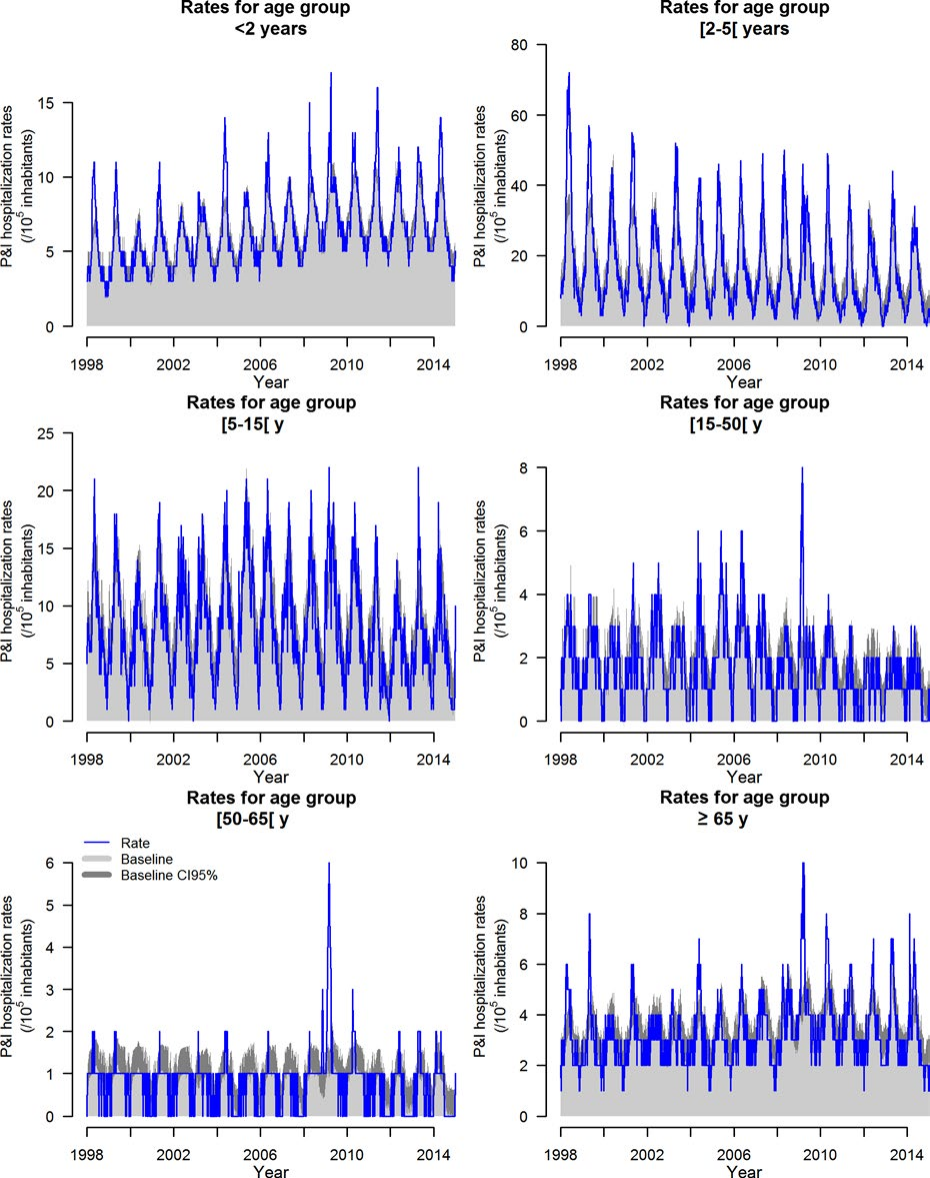
Weekly P&I hospitalization rate per 100 000 inhabitants and the respective estimated baseline and baseline upper limit in the absence of the impact of influenza epidemics from 1998 to 2015

Excess hospitalization rates were calculated by subtracting P&I weekly hospitalization rates baseline, obtained through the model fitting, from the observed weekly P&I hospitalization rates during influenza epidemic periods (Figure 1).

Season excess P&I hospitalization rates 95% confidence level were calculated by approximation to the normal distribution, using as standard error the product of the square root of the number of weeks with excess mortality by the standard deviation of the model residual.

Correlation between season excess rate of hospitalizations and influenza vaccine coverage (last column of Table 1) was measured with Pearson and Spearman correlation coefficient. Confidence interval for coefficient between the vaccine coverage and the season excess P&I hospitalizations rate among the individuals aged 65 years or more was based on bootstrap.^28^, ^29^

**Table 1:** Excess P&I hospitalization rate and absolute number per season and predominant virus type, overall and age group specific. Influenza vaccine coverage in the elderly

Season	Predominant virus	Overall		<2 y		2‐5 y		5‐15 y		15‐50 y		50‐65 y		≥65 y		ICV (%) ≥65 y
		Rate (CI 95%)	Number	Rate (CI 95%)	Number	Rate (CI 95%)	Number	Rate (CI 95%)	Number	Rate (CI 95%)	Number	Rate (CI 95%)	Number	Rate (CI 95%)	Number	
Overall		19.4 (17.8‐20.0)	1932	79.8 (71.3‐83.7)	160	17.5 (13.8‐19.5)	52	5.1 (4.1‐5.7)	53	3.5 (3.0‐3.9)	167	11.2 (9.7‐11.8)	206	68.3 (62.8‐70.7)	1227	
1998/99	A(H3)	35.4 (33.3‐37.4)	3451	276.6 (263.8‐289.3)	588	26.5 (22.5‐30.6)	81	2.7 (1.9‐3.6)	29	1.8 (1.3‐2.4)	90	21.1 (19.1‐23.1)	351	157.3 (149.9‐164.8)	2475	31.3
1999/00	A(H3)	25.7 (23.9‐27.5)	2529	160.8 (150.0‐171.7)	343	17.1 (13.1‐21.2)	52	7.9 (6.5‐9.2)	83	0.0	0	18.3 (16.5‐20.0)	308	93.9 (87.7‐100.2)	1525	39.0
2000/01	B	0.0	0	44.5 (36.8‐52.2)	95	0.0	0	0.0	0	0.0	0	0.0	0	0.0	0	Not available
2001/02	A(H3)	17.9 (16.2‐19.6)	1782	165.6 (154.7‐176.4)	353	23.0 (18.5‐27.6)	73	5.8 (4.7‐6.8)	61	0.0	0	12.5 (10.8‐14.3)	216	67.8 (61.6‐74.1)	1142	41.9
2002/03	B	0.0	0	0.0	0	13.5 (9.9‐17.0)	43	5.3 (4.1‐6.5)	56	0.0	0	0.0	0	0.0	0	36.9
2003/04	A(H3)	18.4 (16.7‐20.1)	1832	0.0	0	22.1 (18.0‐26.1)	71	2.1 (1.3‐3.0)	22	0.0	0	8.6 (7.1‐10.1)	151	87.2 (79.8‐94.7)	1492	47.0
2004/05	A(H3)	46.1 (43.9‐48.3)	4615	93.4 (82.6‐104.3)	192	32.6 (26.9‐38.4)	103	4.4 (3.5‐5.2)	46	6.1 (5.1‐7.1)	297	19.4 (17.3‐21.5)	347	194.9 (186.8‐203.1)	3426	39.0
2005/06^a^	B/AH1	–	–	–	–	–	–	–	–	–	–	–	–	–	–	41.6
2006/07	A(H3)	15.2 (13.8‐16.7)	1530	56.6 (47.2‐66.0)	111	10.9 (8.1‐13.8)	34	10.9 (9.7‐12.1)	114	2.0 (1.5‐2.5)	97	0.0	0	67.8 (62.0‐73.5)	1222	50.4
2007/08	B	2.3 (1.4‐3.2)	228	70.5 (61.9‐79.1)	137	16.1 (12.0‐20.2)	48	7.6 (6.2‐9.1)	80	0.0	0	0.0	0	0.0	0	51.0
2008/09	A(H3)	17.2 (15.7‐18.6)	1726	76.2 (67.6‐84.8)	146	0.0	0	0.0	0	0.0	0	5.9 (4.6‐7.3)	113	74.9 (69.6‐80.2)	1391	53.3
2009/10	A(H1)pdm09	29.3 (27.4‐31.3)	2951	123.2 (113.0‐133.4)	236	47.6 (42.7‐52.6)	140	31.8 (30‐33.6)	331	29.8 (28.7‐30.9)	1419	33.4 (31.5‐35.2)	638	0.0	0	52.2
2010/11	B/A(H1)pdm09	18.6 (16.9‐20.3)	1862	98.3 (88.8‐107.7)	182	33.3 (27.5‐39.0)	94	2.6 (1.8‐3.5)	27	8.5 (7.5‐9.5)	396	21.9 (20.0‐23.8)	428	28.3 (23.6‐33.0)	546	48.3
2011/12	A(H3)	32.5 (30.5‐34.4)	3239	37.2 (29.6‐44.9)	65	0.0	0	0.0	0	0.0	0	8.9 (7.4‐10.4)	176	140.1 (133.8‐146.3)	2748	43.4
2012/13	B/A(H1)pdm09	10.2 (8.5‐11.9)	1007	0.0	0	0.0	0	0.0	0	3.2	143	8.7 (7.4‐10.0)	173	36.2 (30.0‐42.5)	724	44.9
						
2013/14	A(H1)pdm09	11.1 (9.4‐12.8)	1100	74.4 (64.9‐83.8)	117	37.2 (32.2‐42.2)	101	0.0	0	5.2 (4.3‐6.2)	232	5.7 (4.6‐6.8)	114	8.5 (5.1‐11.8)	172	49.9
2014/15	B	31.0 (29.0‐33.0)	3060	0.0	0	0.0	0	0.0	0	0.0	0	14.1 (12.5‐15.7)	282	136.0 (128.6‐143.5)	2766	50.9

a No influenza activity in this season.

A significance level of 5% was considered.

## 3 RESULTS

### 3.1 Overall burden of influenza epidemics

A total of 395 079 P&I hospitalizations were registered between weeks 40/1998 and 40/2015. The seasonal average P&I excess hospitalizations associated with influenza epidemics was 1932 (range 0‐4615). This absolute value represented an average seasonal excess P&I hospitalization rate of 19.4 per 100 000 inhabitants (range 0‐46.1/10^5^).

The analysis of the P&I excess hospitalizations per season (Table 1, overall column) revealed higher absolute excess in 2004/2005 (excess rate of 46.1 per 100.000 inhabitants). On the other hand, in three seasons (2000/2001, 2002/2003 and 2005/2006) no excess P&I hospitalizations were estimated.

Seasons with influenza A(H3) predominance presented the higher average excess absolute number and rate. Looking to the excess rank, the first three positions were occupied by seasons with A(H3) subtype predominance and the co‐predominance of B and A(H3) occupied the forth. In the fifth position was the pandemic A(H1)pdm09. Seasons with influenza B predominance were the ones with the lowest impact. Average excess P&I hospitalization rates associated with influenza epidemics with A(H3) predominance were 3.3 and 1.4 times the average excess P&I hospitalization rate estimated during the influenza B predominant or co‐predominant seasons and the influenza A(H1)pdm09 predominant seasons, respectively.

### 3.2 Age‐specific estimates

**Figure 2:**
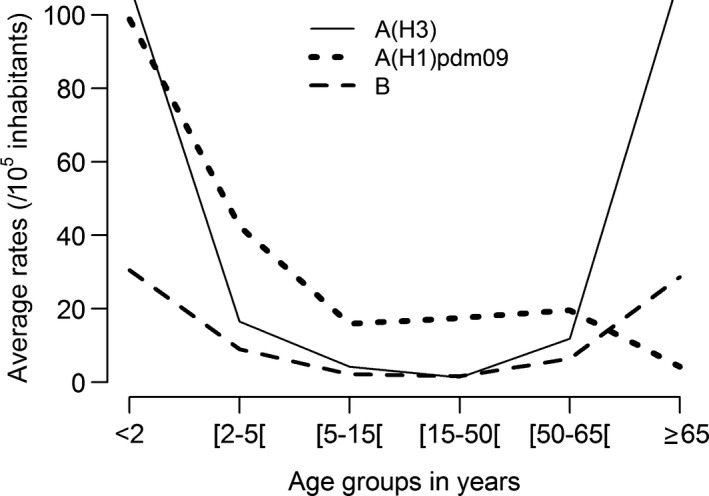
Average excess P&I hospitalization rates per predominant (sub)‐type of influenza virus by age group

**Figure 3:**
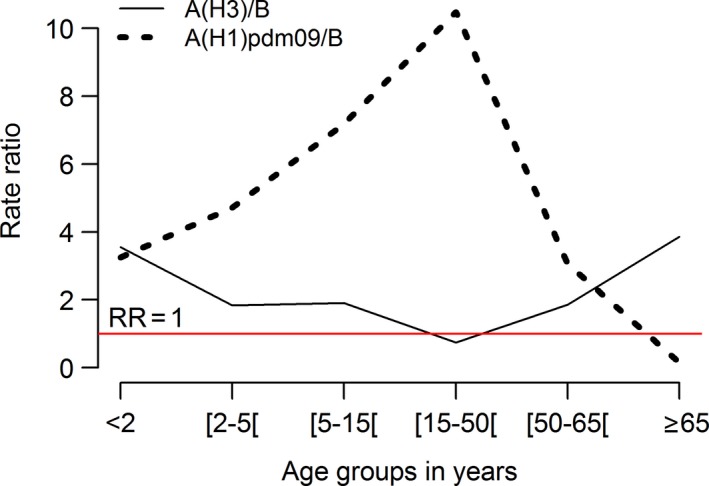
Average excess P&I hospitalization rates ratios for A(H3) and A(H1)pdm09 predominant (sub)‐type of influenza virus compared to B predominant seasons by age group

The analysis of the influenza‐associated P&I excess hospitalizations distribution by age group, season, and predominant (sub)‐type virus type (Table 1) revealed high heterogeneity. Statistically significant influenza‐associated P&I excess hospitalizations were observed in all age groups but not for all seasons. The more extreme age groups (<2 and ≥65 years) presented higher impacts on seasons with A(H3) predominance (Figure 2). This profile was similar to the observed during influenza B predominant seasons. The average excess P&I hospitalization rates for A(H3) seasons was higher than influenza B seasons for all age groups with exception for those in the 15‐50 age group (Figure 3).

**Figure 4:**
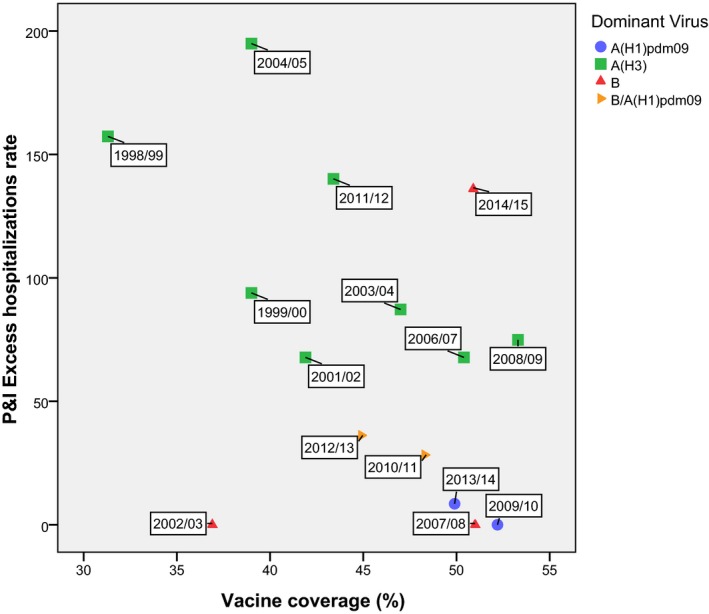
Scatter plot of the vaccine coverage rates vs the excess P&I hospitalizations rate according to the dominant subtype virus for seasons 1998/1999‐2014/2015, on individuals aged 65 y or more

A very different distribution of the excess P&I hospitalization rate was registered for the influenza A(H1)pdm09 predominant seasons, the highest rate being observed for the <2‐year age group. Considering the age group for which the vaccine is recommended (≥65 years), the lowest average excess P&I hospitalization rate, per predominant (sub)‐type influenza season, was observed during the pandemic season (Figure 4). Nevertheless, for the individuals with age between 5 and 64 years it was during the pandemic that the highest excess P&I hospitalization rates were observed in particular for the age group 5‐49 in which the rate was 10 times the average rate for the influenza B predominant seasons.

### 3.3 Association between influenza vaccine coverage and seasonal excess P&I influenza‐associated hospitalization rates among the elderly

The overall correlation between the influenza vaccine coverage and the seasonal P&I excess hospitalizations rate was −0.381 (95% CI: −0.750 to 0.226) measured with Pearson correlation and −0.306 (95% CI: −0.766 to 0.332) with the Spearmen correlation. When the analysis was restricted to seasons with influenza A(H3) predominance, the correlation coefficient was stronger (−0.653; 95% CI: −0.950 to −0.137 using Pearson and −0.675; 95% CI: −1.000 to 0.025 with Spearman).

## 4 DISCUSSION

The present study is the first Portuguese comprehensive analysis aimed at estimating the excess of pneumonia and influenza hospitalizations associated with influenza epidemics, using a wide period of analysis that encompasses 17 influenza seasons. This study estimated an average of 1932 seasonal excess P&I hospitalizations associated with influenza epidemics. This estimate varied widely across the 17 seasons from no excess (in 2000/2001, 2002/2003, and 2005/2006 seasons) to a maximum of 4615 excess P&I hospitalizations (2004/05 season). Taking into account the population, these estimates represented a seasonal excess P&I hospitalizations rate of 19.4 per 100 000 habitants reaching a maximum of 46.1 per 100 000 habitants. Our overall estimated P&I influenza‐associated excess hospitalization rate was in the agreement with the reported estimates in other studies.^30^

Overall, seasonal P&I excess hospitalization rates were higher among the individuals with <2 years and among those with 65 or more years of age. These results are compatible with those published in other studies that include these two groups at higher risk of being hospitalized during influenza seasonal epidemics.^3^, ^31^, ^32^ Nevertheless, this pattern was not equal in all seasons, specifically during the pandemic A(H1)pdm09 season, where no excess P&I hospitalizations were observed among the elderly (≥65 years). On the other hand, the excess P&I hospitalization was the highest observed within the individuals aged between 2 and 65 years, a population group that, during seasonal influenza epidemics, presented the lowest influenza‐associated excess P&I hospitalization rates. This observation was consistent with other European studies^30^, ^33^, ^34^, ^35^ and with mortality profile of the pandemic A(H1)pdm09 in Portugal.^22^ According to some authors,^36^, ^37^ the elderly population was largely spared during the pandemic mainly due to their past exposure to influenza virus similar to the A(H1N1)pdm09 virus and younger age groups were naive to this new circulating virus. This fact was reflected in the recommendations issued by health authorities regarding the pandemic monovalent vaccine uptake that did not include the individuals aged 65 or more years of age.^38^, ^39^

The excess P&I hospitalizations profile was maintained in seasons with predominance circulation of A(H3) and B virus. Seasons with A(H3) influenza virus subtype predominance presented on average a higher excess P&I influenza‐associated hospitalization rate, among the extreme age groups. Comparing with seasons with other virological profile, A(H3) predominant seasons, presented a P&I excess hospitalization rate, among the younger (<2 years), very similar to the A(H1)pdm09 age group but higher than B or B/A(H1)pdm09 seasons. A(H3) also had much higher impact on the elderly age group compared to the other virus profiles. This result has been largely described in other study,^5^ targeting these two age groups as the ones with the highest impact of influenza‐associated hospitalizations and in particular when influenza A(H3) subtype is predominant. The previously described excess P&I mortality rate profile in Portugal^8^ has showed a different profile, with small or no impact among the younger age groups contrasting with higher excess P&I mortality rate for the age groups above 65 years or more. These results are in line with an expected higher influenza lethality rate among the older age groups.

In this study, among the individuals with 65 or more years of age, a negative correlation was observed between the seasonal influenza vaccine coverage and seasonal P&I excess hospitalizations during the influenza epidemics. This negative correlation was not statistical significant when all seasons were considered (Pearson correlation = −0.381); however, statistical significance was observed when considering only seasons with influenza A(H3) subtype predominance (Pearson correlation = −0.653). One possible reason for the increased strength in the correlation can be related to fact that the other seasons had a low impact in this age group. When considering all seasons, an increased dispersion was observed, reducing the strength correlation. Among the elderly (≥65 years), a negative correlation was also previously observed in another study that measured the correlation between vaccine coverage and seasonal ILI attack rates, and published a spearman rank coefficient of −0.359 and −0.899, respectively, for all seasons and only for the A(H3) predominant.^19^ In 2016, a study that included 14 European Union countries, including Portugal,^40^ has analyzed the correlation between seasonal ILI incidence rates and vaccine coverage among all the population (except for individuals with 65 or more years of age) and showed no consistent results between the countries, identifying only negative correlation among the elderly population for England (−0.80), the Netherlands (−0.60), and Germany (−0.57). For Portugal, the study included seasons 1998‐1999 to 2013‐2014 showing correlation coefficients different from those obtained in our previous study that included seasons 1998‐1999 to 2006‐2007.^19^ A possible explanation for the difference may be the data source for ILI rates, that is, the use of provisory ILI rates (in the European study) and definitive rates calculated in the end of the season (in the national study).

The results presented in the this study must be interpreted in light of the methods and data limitations. A time series ecological method was used to estimate the seasonal P&I excess hospitalization rates in the study period. As such, the excess P&I hospitalization rate cannot be considered fully attributable to influenza epidemics but only associated with the occurrence of the epidemics. Other covariates as the circulation of other respiratory virus, like the respiratory syncytial virus, were not considered in the model. This fact could have overestimated the impact of the influenza epidemics reported in the present report, mainly for the younger age groups (<2 years).^41^, ^42^ Nevertheless, when stratified by influenza virological profile, there were differences between A(H3), A(H1)pmd09, or B predominant seasons, data consistent with previous reports, which suggests that P&I excess hospitalizations were sensible and probably specific in capturing the impact of the influenza epidemics. In this study, an ARIMA model was used and no information about virus type or subtype circulation was considered. Unlike previous studies,^12^, ^42^ the weekly distribution of the influenza virus detection was not included as covariate in the model. This fact did not allow for estimation of the excess P&I influenza‐associated hospitalization by (sub)‐type influenza virus. However, according to Thompson et al,^10^ similar influenza‐associated mortality was obtained using ARIMA or Poisson models that included influenza type as covariate. This study also highlighted that, in the absence of robust covariates data, namely on weekly influenza type and subtype data in specific age groups, ARIMA models are a good candidate model to be used in influenza‐associated excess studies.^10^

Finally, our study did not take into consideration the correlation between circulating influenza virus and vaccine strains. This could have implications on the correlation estimates between vaccine coverage and influenza‐associated P&I hospitalizations.

In summary, our type/subtype and age‐specific influenza‐associated P&I hospitalizations are in accordance with the literature on influenza excess hospitalization and provide for the first time a measure of the impact of influenza in Portugal for a wide period. Also, this study evidences that there is a negative correlation between influenza vaccination and the influenza‐associated P&I hospitalizations, in particular when A(H3) is circulating and in the risk group of the elderly for which the vaccine is recommended. These results are encouraging, specially to validate campaigns for vaccine uptake in seasonal epidemics and in specific age groups in a future pandemic. Nevertheless, more studies should be performed to evaluate the consistency of these results. Following this line of thought, it is important to continue researches in Portugal by looking into other hospitalizations causes and by incorporating additional information on influenza type/subtype circulation as well as other respiratory virus as a way to fortify these findings.
