# Supplementary material for: Excess pneumonia and influenza hospitalizations associated with influenza epidemics in Portugal from season 1998/1999 to 2014/2015
Source: Influenza Other Respir Viruses. 2018 Feb 19;12(1):153–60. doi: 10.1111/irv.12501 (PMC5818339; doi:10.1111/irv.12501)
Supplement: Supplementary file 1 [file IRV-12-153-s001.docx]

**Supplement Table 1:** Events potentially associated with excess hospitalizations observed in the period from week 26 of 1998 to week 27 of 2010 in Portugal

| Event | Period (week/year) | Number of weeks |
| --- | --- | --- |
| 1998-1999 influenza epidemic | 52/1998 to 9/1999 | 11 |
| 1999-2000 influenza epidemic | 1 to 8/2000 | 8 |
| 2000-2001 influenza epidemic | 3 to 6/2001 | 4 |
| 2001-2002 influenza epidemic | 1 to 11/2002 | 11 |
| 2002-2003 influenza epidemic | 48/2002 to 01/2003 | 6 |
| 2003 heat wave | 25 to 26 and 31to 35/2003 | 7 |
| 2003-2004 influenza epidemic | 44/2003 to 1/2004 | 10 |
| 2004/2005 influenza epidemic | 53/2004 to 11/2005 | 12 |
| 2006-2007 influenza epidemic | 3 to 9/2007 | 7 |
| 2007 heat wave | 30 to 32/2007 | 3 |
| 2007-2008 influenza epidemic | 3 to 8/2008 | 6 |
| 2008 heat wave | 29 to 30/2008 | 2 |
| 2008-2009 influenza epidemic | 50/2008 to 4/2009 | 7 |
| 2009 pandemic | 22 to 37/2009 | 16 |
| 2009-2010 influenza epidemic | 44 to 52/2009 | 9 |
| 2010 heat wave | 27 to 33 and 35 to 38/2010 | 11 |
| 2010-2011 influenza epidemic | 50/2010 to 6/2011 | 9 |
| 2011 heat wave | 25 to 26, and 30 to 34/2011 | 7 |
| 2011-2012 influenza epidemic | 4 to 13/2012 | 10 |
| 2012 heat wave | 26 to 27, 29 to 30 and 32 to 33/2012 | 6 |
| 2012-2013 influenza epidemic | 4 to 13/2013 | 10 |
| 2013 heat wave | 26 to 29 and 32 to 39/2013 | 12 |
| 2013-2014 influenza epidemic | 3 to 9/2014 | 7 |
| 2014 heat wave | 24 to 25/2014 | 2 |
| 2014-2015 influenza epidemic | 1 to 10/2015 | 10 |
| 2015 heat wave | 25 to 26/2015 | 2 |

**Supplement Table 2.** Seasonal ARIMA best-fitted models by R package forecast and Box-Ljong test for residuals auto correlation

| Age groups | Model | Box-Ljung test for auto-correlation of residuals |
| --- | --- | --- |
| Overall | ARIMA(2,1,1)(2,0,2)[52] | X = 1.7901, df = 6.789, p = 0.9654 |
| <2 | ARIMA(2,1,2)(2,0,2)[52] | X = 14.4699, df= 6.789, p = 0.03886 |
| 2-4 | ARIMA(1,1,1)(1,0,1)[52] | X = 15.736, df= 6.789, p = 0.02455 |
| 5-14 | ARIMA(3,1,1)(2,0,2)[52] with drift | X = 10.8705, df= 6.789, p = 0.1323 |
| 15-49 | ARIMA(2,1,2)(0,0,2)[52] | X = 11.9567, df= 6.789, p = 0.09275 |
| 50-64 | ARIMA(1,1,3)(0,0,2)[52] | X = 15.3473, df= 6.789, p = 0.02830 |
| >= 65 | ARIMA(2,1,2)(2,0,2)[52] with drift | X = 22.864, df= 6.789, p = 0.001545 |
